# Socio-economic determinants for malaria transmission risk in an endemic primary health centre in Assam, India

**DOI:** 10.1186/2049-9957-3-19

**Published:** 2014-06-24

**Authors:** Kavita Yadav, Sunil Dhiman, Bipul Rabha, PK Saikia, Vijay Veer

**Affiliations:** 1 Defence Research Laboratory, Tezpur, Assam 784001, India; 2 Department of Zoology, Gauhati University, Guwahati, India

**Keywords:** Malaria risk factors, Socio-economic, Awareness, Udalguri, Assam

## Abstract

**Background:**

Malaria is a major cause of morbidity and mortality in Northeast India. As there is limited information available on the potential influence of socio-economic variables on malaria risk, the present study was conducted to assess the influence of demographic factors, the socio-economic status, and knowledge, awareness and education on malaria occurrence.

**Methods:**

Demographics, malaria knowledge and socio-economic variables were collected in four randomly selected health sub-centres of the Orang primary health centre in the Udalguri district, Assam and the association of malaria occurrence with different variables were analysed. The trend of malaria occurrence for different income groups, proximity to health centres and number of mosquito bites per day was also determined using the chi-square test. Relative risk (RR) for gender, house type, knowledge and use of bed nets was determined using Katz approximation.

**Results:**

Out of the 71 household heads interviewed, 70.4% (50/71) were males. About half (54.9%, 39/71) of the participants had a history of malaria in the last two years, of which 64.1% (25/39) were males, while 35.9% (14/39) were females (χ^2^ = 5.13; p = 0.02; RR = 1.79). Of the total population surveyed, 49.3% lived in bamboo houses and 35.2% lived at a distance of >3 km from the nearest health centre. The number of participants who had a history of malaria decreased with an increasing monthly income (p < 0.0001). Malaria occurrence was higher among the households living in bamboo houses (69.2%), as compared to Kucha houses (20.5%) and Pucca houses (10.3%). No significant association was observed between education level and malaria occurrence (p = 0.93). The participants who did not use bed nets regularly reported a high occurrence of malaria infection as compared to those who used bed nets everyday (p < 0.0001).

**Conclusions:**

Lower income, house type, distance to health sub-centre, knowledge and awareness about malaria, number of mosquito bites per day and use of bed nets were positively associated with malaria occurrence. Increasing the number of health sub-centres close to rural areas, improving the economic status and increasing awareness about malaria prevention measures will thus help to reduce malaria-associated morbidities.

## Multilingual abstracts

Please see Additional file 1 for translations of the abstract into the six official working languages of the United Nations.

## Background

According to the World Health Organization’s (WHO’s) latest estimates, about 207 million malaria cases (range: 135–287) and approximately 0.63 million deaths (range: 0.47–0.79) were reported in 2012 [1]. Malaria has a higher inequality in distribution than any other disease of public health importance, as about 58% of malaria deaths occur among the poorest 20% of the world's population [2]. Endemic malaria results in tremendous economic losses annually and is a central element of the vicious cycle of poverty in many developing countries [3,4]. The global malaria control strategy has largely emphasised malaria control by insecticide-treated bed nets (ITNs), including long-lasting insecticidal nets (LLINs), indoor residual spraying (IRS), increased case detection and subsequent treatment, as well as other vector control interventions, but comparatively less emphasis has been given to the socio-economic depression complex from which people have difficulty singling out malaria for various concerns [1,5]. The people living in malaria endemic countries with many other scourges, such as hunger, poverty and other diseases cannot understand why they would target malaria rather than make efforts to eliminate poverty and hunger. Therefore, in addition to strengthening the healthcare system, the success of malaria control depends significantly upon the knowledge and socio-economic status of the affected populations in the malaria endemic countries. Malaria is frequently referred to as a disease of the poor and the economic status of populations at risk has proved to be a great challenge in malaria control programmes [6]. Malaria-associated morbidity and the cost of treatment are important burdens, and create barriers for the overall social and economic development.

There is a high incidence of malaria in the northeastern states of India, which account for >20% of the deaths that are reported in the country annually [7-10]. People live under low socio-economic conditions and have high levels of immunity enabling them to serve as reservoirs for malaria transmission [11]. Poor socio-economic conditions, knowledge and perception about malaria and antimalarial policies have contributed to widespread malaria throughout the region [8,9,12-14]. Similar to other malaria endemic countries, personal protection using ITNs and malaria treatment with an effective antimalarial are the main strategies against malaria in India (http://www.nvbdcp.gov.in/). The characteristics of the rural environment may also make malaria control difficult due to a high ethnic population density, the geopolitical context and an unfocused nature of malaria vectors breeding sites, which all present major challenges to the integrated malaria control strategies [7,8,15]. Various socio-demographic factors, such as ethnic groups, parents’ education levels and occupation, use of personal protective measures and family living standards are important risk factors for malaria transmission and epidemics [16].

Knowledge of malaria and socio-economic upliftment are key factors in adopting the appropriate intervention strategies. Keeping in mind that there is a large number of risk factors that influence vulnerability to malaria including proper knowledge about malaria transmission and prevention, demography and socio-economic status of different population groups, the present study was undertaken to identify factors predisposing to malaria in a highly endemic primary health centre in the Udalguri district, Assam, India.

## Methods

### Study area

**Figure 1:**
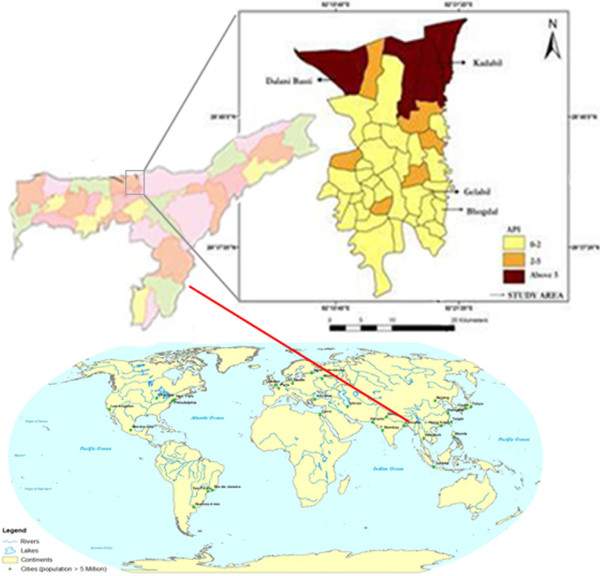
Annual parasitic index (API) of the health sub-centres of the Orang primary health centre during 2010–11 (colour indicates the level of API).

The Orang primary health centre, situated between 92° 10’ 40” E longitude to 92° 21’ 20” N latitude on the north bank of river Brahmaputra at 105.2 metres above sea level (see Figure 1), is dominated by ethnic tribes of Bodo, Adivasi and Rabha. Low literacy rates, poverty and reluctance to accept medical treatment are common features of these villagers. The climate is humid with semi-dry hot summers and cold winters. The annual rainfall, temperature and humidity of the area ranges from 1500 to 2000 mm, 13.5–34.5°C and 82–88%, respectively. Malaria is endemic in the entire Orang primary health centre and the majority of the cases fall into the category of *Plasmodium falciparum* malaria. Malaria transmission is perennial and uninterruptedly supported by malaria vectors, *Anopheles dirus, An. minimus, An. fluviatilis, An. annularis* and *An. culicifacies*[11,12,14]. The study area is covered with tea gardens, includes the Rajiv Gandhi Orang National Park and has many small rivers and streams. The primary health centre studied consists of 42 health sub-centres covering a population of around 250,000 people. From a climatic point of view, the study region is conducive to malaria epidemics.

### Study design

A cross-sectional study was carried out from March to September 2012 in four randomly selected health sub-centres, namely Kadabil, Dhalanibasti, Gelabil and Bhogdol in the Orang primary health centre, while assuming that all the health sub-centres are homogenous and have a similar malaria situation. During the surveys, about 900 houses were visited to ascertain their willingness to participate in the study, and thereafter a total of 71 household heads representing 385 individual household members were interviewed. In the remaining houses, people were not willing to participate in the study due to one or more of the following reasons: religious beliefs, absence of house head or a feeling that it is not worth participating. Each house represented a family and a household represented the total family members, whereas the head of a household was the person who was perceived to be the primary decision maker in the family. Although the sample size in the present study is small, it is still very useful to take it into consideration when undertaking malaria control efforts in a comparatively inaccessible malaria endemic study area.

### Data collection

Details about personal attributes, knowledge of malaria and socio-economic parameters were recorded in a structured questionnaire that was printed in English and Assamese (local language). A local health worker, in the presence of the community head, recorded the responses of each individual interviewed by the investigators. The community head and the local health worker are the most respected and trustworthy persons in the community and were therefore involved in the study. Specific variables recorded included: age, gender, education level, knowledge about malaria transmission and prevention, house type, monthly income, other diseases besides malaria, mosquito net use, whether the mosquito net used is the long-lasting insecticidal nets (LLIN)/ITN, frequency of bed net use and source of obtaining bed net, proximity to a health centre and approximate number of mosquito bites per day. The responses of the participants were later verified by their records held at the health sub-centres. Malaria knowledge was ascertained by asking the following questions:

1. What is malaria?

2. How is malaria caused?

3. How does malaria spread?

4. How can malaria be avoided?

5. What is the treatment for malaria?

6. What is the government doing about malaria?

7. Do you know that the government provides bed nets free of charge and antimalarial treatment is available free of charge as well in the government health centres?

All seven questions included in the present study were carefully selected and are directly related to malaria aetiology, transmission and prevention. The participants who responded well to all questions were defined as having a good knowledge, those who responded to at least five questions were defined as having an adequate knowledge, while those who could not answer more than three questions were considered to have a poor knowledge. The participants who have monthly incomes of <2000 INR (32.14 $) were recorded as poor, those having 2000–5000 INR (32.14–80.36 $) were recorded as average, while those having a monthly income of >5000 INR (80.36 $) were recorded as the high-income group. Monthly incomes of the participants were ascertained after asking them about their assets, agriculture produce and other income. In addition to recording the data, in-depth verbal interviews and group discussions were conducted with the local people aggregated during the study, in order to further extrapolate on the findings recorded on the questionnaire sheets. The present study was a part of a larger study being conducted in the region to assess antimalarial resistance and human-host immune response. The study project was sanctioned by the Department of Defence Research and Development, Government of India and approved by the Institutional Ethical Committee (IEC). Written consent was obtained from the study participants before interviews and all data collected was kept confidential.

### Statistical analysis

Association of malaria occurrence with gender, poverty, house type, knowledge, education, proximity to a health centre, use of bed nets, mosquito bites per day and other awareness parameters were analysed using the chi-square test. Similarly, the trend of malaria occurrence for different income groups, proximity to a health centre and mosquito bites per day was also determined using the chi-square test. Relative risk (RR) for gender, house type, knowledge and use of bed nets was determined using Katz approximation. Odds ratios (OR) for the type of bed net used, knowledge about LLINs and supply of bed nets were determined using Wolf approximation.

## Results

### The malaria situation and socio-demographic characterisation

The spatial distribution of malaria prevalence data of all 42 health sub-centres of the Orang primary health centre collected from the state health authority indicated that the API ranged from 0–5 during 2012. Four health sub-centres (9.5%) had an API of >5, while in six (14.3%) the API ranged from 2 to 5 (see Figure 1). Out of the total 71 household heads interviewed, 70.4% were males and 29.6% were females. The average age of the participants was 36.1 ± 1.7 (mean ± SEM) (range = 20–65; 95% CI = 33.1–39.1), whereas the median number of members per household was five (range = 2–12) (mean ± SEM = 5.4 ± 0.3; 95% CI = 4.9–5.9) (see Table 1). Of the total participants, 54.9% (39/71) had a history of malaria in the last two years, of which 64.1% (25/39) were males and 35.9% (14/39) were females (χ^2^ = 5.13; p = 0.02; RR = 1.79; 95% CI = 1.10–2.89). The education level of 76.1% (54/71) participants was below primary, and only 9.9% (7/71) had a good knowledge of malaria. At least 46.5% (33/71) were poor, 49.3% (35/71) lived in bamboo houses and 35.2% (25/71) had their nearest dispensary at a distance of >3 km (see Table 2). Most participants (76.1%) reported having > five mosquito bites every day, 70.4% (50/71) reported daily bed net use and 50.7% (36/71) used LLINs. All the LLINs were provided by the Assam State Government agency, while only 15.5% of the participants were aware about the LLINs (see Table 3).

**Table 1:** Demographic characteristics and their association with malaria history among the study population in the Orang primary health centre

***Characteristic***		***N (%)***	***p (χ***^***2***^ )^*^	***M (%)***	***p (χ***^***2***^ ***)***^***#***^
Age	15–35	33 (46.5)	< 0.0001 (18.5)	25 (75.8)	0.004 (11.7)
	36–55	28 (39.4)		11 (39.3)	
	56 & above	10 (14.1)		3 (30.0)	
Sex	Male	50 (70.4)	< 0.0001 (23.7)	25 (50.0)	0.3 (1.1)
	Female	21 (29.6)		14 (66.7)	
Body Complexion	Dark	29 (40.8)	0.02 (4.1)	22 (75.9)	0.007 (7.3)
	Fair	42 (59.2)		17 (40.5)	
Body Odour	Strong	44 (62.0)	0.04 (7.2)	27 (61.4)	0.25 (1.3)
	Mild	27 (38.0)		12 (44.4)	
Body Clothing	>50%	64 (90.1)	< 0.0001 (88.3)	34 (54.7)	0.6 (0.3)
	<50%	7 (9.9)		5 (71.4)	
Marital Status	Married	63 (88.7)	< 0.0001 (82.3)	33 (52.4)	0.4 (0.7)
	UM	8 (11.3)		6 (75.0)	
Family Size	1–3	16 (22.5)	< 0.0001 (19.6)	9 (56.3)	0.6 (1.0)
	4–6	38 (53.5)		19 (50.0)	
	7 & above	17 (23.9)		11 (64.8)	

N – total number of participants; M – participants with a history of malaria.

*for characteristic variables; ^#^for malaria history.

**Table 2:** Socio-economic characteristics of the study population in the Orang primary health centre

***Characteristics***		***N (%)***	***p (χ***^***2***^ ***)***
Education	Primary	54 (76.1)	< 0.0001 (36.5)
	Above Primary	17 (23.9)	
Monthly Income in Indian Rupee	<2000 (32.14 $)	33 (46.5)	< 0.0001 (33.1)
	2000–5000 (32.14–80.36 $)	33 (46.5)	
	>5000 (80.36 $)	5 (7.0)	
House Type	Bamboo	35 (49.3)	< 0.0001 (30.5)
	Kucha	30 (42.3)	
	Pucca	6 (8.5)	
Information Source	Newspaper	3 (4.2)	< 0.0001 (50.9)
	TV/Radio	43 (60.6)	
	None	25 (35.2)	
Distance to Nearest Health Centre	<1 Km	31 (43.7)	0.02 (8.3)
	1–3 Km	15 (21.1)	
	>3 Km	25 (35.2)	

N – total number of participants.

**Table 3:** Malaria epidemiology related characteristics of the study population in the Orang primary health centre

***Characteristics***		***N (%)***	***p (χ***^***2***^ ***)***
Knowledge About Malaria	Poor	31 (43.7)	< 0.0001 (26.5)
	Adequate	33 (46.5)	
	Good	7 (9.9)	
Water Source Near House	River	8 (11.3)	< 0.0001 (112.4)
	Nallah	5 (7.0)	
	Well/Tank	58 (81.7)	
Bed Net Use	Daily	50 (70.4)	< 0.0001 (79.9)
	Mostly	21 (29.6)	
	Never	0 (0.0)	
Bed Net Type	LLIN	36 (50.7)	0.87 (0.03)
	ITN	35 (49.3)	
Source of Bed Net	Govt.	36 (50.7)	0.87 (0.03)
	Own	35 (49.3)	

N – total number of participants.

### Risk factor analysis for malaria occurrence

In the univariate analysis, significant associations were observed between malaria infection and selected socio-demographic characteristics of the study participants. The occurrence of malaria was found to be statically similar among both sexes (χ^2^ = 1.1; p = 0.3). Significantly more malaria cases were observed among poor people (71.8%) who had a monthly income of <2000 INR as compared to those who had a monthly income of >2000 INR (χ^2^ = 24.2; p < 0.0001). Malaria cases decreased with an increasing monthly income (p < 0.0001). House type was significantly associated with malaria occurrence (χ^2^ = 17.0; p = 0.0002). Malaria was reported to be prevalent among 69.2% (27/39) of the households living in bamboo houses, as compared to 20.5% living in Kucha houses (8/39) and 10.3% in Pucca houses (4/39). Knowledge and awareness of malaria significantly influenced the malaria occurrence among the participants. Malaria was prevalent among 69.2% (27/39) of the participants who had a poor knowledge about malaria (χ^2^ = 25.5; p < 0.0001). Distance to the nearest dispensary was significantly associated with malaria occurrence as well. Malaria was observed to be higher among those who needed to travel more to assess the nearest health facility (χ^2^ = 25.0; p < 0.0001). There was a significant linear trend between malaria occurrence and distance to a health facility as more cases were observed among the households who had the nearest health facility at a distance of >3 km (χ^2^ = 24.7; p < 0.0001).

Although 76.1% (54/71) of the participants who had malaria also had a below primary education level, there was no significant association between the education level of the participants and the occurrence of malaria (χ^2^ = 0.008; p < 0.93; RR = 0.91; 95% CI = 0.57–1.46). The number of mosquito bites was significantly associated with malaria occurrence among the participants. Malaria occurrence was observed among all the participants who reported to receive >10 mosquito bites, as compared to those who received <10 mosquito bites per day. The difference was statically significant (χ^2^ = 10.1; p < 0.006), and a significant linear trend was observed between malaria occurrence and the number of mosquito bites per day (χ^2^ = 9.7; p < 0.001). Use of bed net was associated with malaria as the number of participants who did not use bed nets regularly reported a high occurrence of malaria infection as compared to those who used bed nets daily (χ^2^ = 17.33; p < 0.0001). There was a significant association observed between the supply and type of bed nets used and the occurrence of malaria. Among the participants who reported malaria, 79.5% (31/39) used LLINs as compared to the ITNs (χ^2^ = 7.6; p < 0.003; OR = 0.27 (95% CI = 0.11–0.66).

## Discussion

Although substantial progress has been made in controlling malaria, approximately 1.3 billion people continue to be at risk in the Southeast Asian region. Ten of the eleven member nations of the WHO-SEA region are malaria endemic, contributing 15% to the global malaria burden [17]. In addition to killing thousands of people, malaria is an enervating disease that creates a massive socio-economic burden globally, mainly for poor people living in areas with limited access to health care [18]. The Udalguri district is a malaria prone area, reporting API of >10 in the majority of the health sub-centres in recent years [9]. An estimated 54% of the population of the Udalguri district lives below the poverty line and is dominated by tribal communities. The tribal people benefit the least from the socio-economic development and still prefer to use traditional systems to treat diseases [19].

The results of malaria occurrence among both sexes were similar to the previous studies conducted in the region [7,8,20]. However, some studies have suggested that females are more vulnerable to malaria as they are in charge of many household activities, which cause them to be at a greater risk of acquiring the malaria infection [21]. Poverty was associated with malaria occurrence in the study population. Many studies have regarded malaria as a disease of the poor, which is substantiated by the fact that the malaria burden is often concentrated in the poorest continents and countries [21-25]. The poor have comparatively less access to antimalarials and anti-mosquito measures, since they cannot afford personal protection measures, a clean environment free of mosquito breeding sites, and are particularly vulnerable to the impact of ineffective diagnosis and treatment due to financial and cultural implications. Further, the poor population is often heavily marginalised by the health sector and survives under tremendous burden of malaria and other communicable diseases [26]. Bamboo houses and a greater number of mosquito bites per day were positively associated with a history of malaria. It is most likely that poorly constructed bamboo houses might have a number of gaps and holes through which a vector mosquito could easily enter following the scent of human hosts. A recent study in Laos suggested that good quality houses could reduce disease transmission by reducing the human-mosquito contact [27].

There was a strong association between common knowledge and awareness about malaria and occurrence of malaria among the study participants. The participants who satisfactorily responded to the questions about malaria transmission and prevention were less affected by malaria as compared to those who had a poor knowledge. On the other hand, the level of education of the participants did not have any impact on the occurrence of malaria. A previous study conducted in Lagos also indicated that education was not significantly associated with malaria infection among pregnant women [28]. Community knowledge and awareness has a significant influence on malaria control, and individual knowledge, awareness and beliefs may also affect malaria occurrence. The misconception and beliefs about malaria among some communities continuously battle with the correct scientific information and pose major setbacks in malaria control efforts [3,29,30].

Distance from the house to the nearest health facility was another risk factor for malaria occurrence. Many previous similar studies have reported that distance to health centres influenced the treatment seeking behaviour of individuals, compounding the malaria situation [31]. The distance to a health centre could be regarded as a factor for health facility accessibility for which the cost of reaching a health centre is met by the patients. A problematic distance to a health centre may result in a household not assessing the health facility frequently due to the cost and loss of work time. Our findings suggest that household members whose houses are close to health centres have more and more timely access to health care as compared to those whose houses are far away from health centres. Further, the households who live closer to health centres have more knowledge and awareness as they have more interaction with the health staff.

We found that the number of mosquito bites received by participants influenced malaria occurrence. The higher the number of mosquito bites per day, the greater the risk of malaria infection [32]. Use of the bed nets was found to be associated with malaria occurrence as well. The households who used bed nets frequently, but not daily were more affected by malaria as compared to those who used bed nets every night. Although, the bed nets were available in every house in the study area, the number was probably still insufficient to achieve total coverage. Therefore, each household did not use bed nets regularly. Further, malaria occurrence was found to be higher among those who used LLINs as compared to those who used ITNs. Many studies have concluded that ITN coverage was related to malaria control knowledge, significantly reduced malaria and was associated with a malaria risk-protective effect [31,33-37]. Although LLINs have been recommended widely as they remain effective for several years, ITNs have also proved to be effective in reducing all causes of malaria mortality and morbidity [33,37]. Insecticide-treated bed nets (ITNs) are easy to use and require less capital outlay as compared to the LLINs, and this might lead to a widespread use of ITNs on a large scale. Studies have indicated that LLINs get damaged at a faster rate in operational conditions and holes and other entry places for mosquitoes are more easily formed. Moreover, torn and improperly used LLINs allow vector mosquitoes to enter and bite the user [38]. Insecticide-treated bed nets (ITNs) have also been shown to provide substantial protection against malaria, however the sustainable use of ITNs needs regular insecticide re-treatment as they lose efficacy after some time [39].

Although malaria distribution is mostly determined by the climatic and environmental factors which affect mosquito and malaria parasite reproduction and proliferation at a given time, malaria is also influenced by various socio-economic factors [3,4,26,28,40,41]. In the current study, important associations between malaria occurrence and risk factors were observed which cannot be explained by any of the factors included in this analysis. Although many factors were associated with malaria occurrence, none of the factors could singly be targeted for comprehensive malaria control. Malaria occurrence is a complex interplay of various factors, which may be region specific and most of which are inter-dependent upon each other. A better understanding of the association between malaria and associated factors is required to design effective policies to tackle malaria. The present study is first of its kind and documents that there are certain key factors which need to be taken into consideration when allocating funds for addressing the malaria problem in the region.

Although the study offers some important findings, it has also some limitations. The study is cross sectional and the sample size is not very large, therefore the possibility of sampling error cannot be overruled. Malaria occurrence was based on the history but not actual testing. Further, the perception of risk and actual malaria incidence vary geographically and seasonally. The study did not involve questions on the re-treatment and age of ITNs. Also, the study used ITNs and LLINs as the sole indicator of control measures, but the usage of IRS, mosquito repellent coils and creams was not assessed.

## Conclusion

In the study area, malaria occurrence was higher among participants who had lower incomes, lived in bamboo houses and were located at a considerable distance from a health sub-centre. Further, knowledge and awareness about malaria, number of mosquito bites per day and use of bed nets were also associated with malaria occurrence. However, the education level of the study participants had no role in malaria occurrence. Although malaria incidence depends on various variables, the present study demonstrates that those variables related to poverty, poor health infrastructure and awareness about malaria are the most important factors in minimising the risk of malaria.

## Competing interest

The authors declare that they have no competing interest.

## Authors’ contributions

KY, PKS and SD conceived the idea. SD, KY and BR collected the data. SD and KY analysed the data. KY prepared the map. SD, BR and KY drafted the manuscript. PKS and VV edited the manuscript and extended technical support. All authors read the manuscript and approved the draft.

## Supplementary Material

Additional file 1 Multilingual abstracts in the six official working languages of the United Nations. Click here for file
