# Supplementary material for: Socio-economic determinants for malaria transmission risk in an endemic primary health centre in Assam, India
Source: Infect Dis Poverty. 2014 Jun 24;3:19. doi: 10.1186/2049-9957-3-19 (PMC4078389; doi:10.1186/2049-9957-3-19)
Supplement: Additional file 1 — Multilingual abstracts in the six official working languages of the United Nations. [file 2049-9957-3-19-S1.pdf]

Translation of the abstract into the six official working languages of the United Nations

المحددات الاجتماعية والاقتصادية لخطر انتقال الملاريا في مركز الرعاية الصحية الأولية للأمراض المستوطنة في ولاية أسام، الهند

كافيتا ياداف، سونيل ديمان ، بيبول رابحة ، بي كيه سيكيا ، فيجاي فير

ملخص

الخلفية: تعتبر الملاريا سببا رئيسيا للإصابة بالأمراض والوفيات في شمال شرق الهند. كما أن هناك معلومات محدودة متوفرة على التأثير المحتمل للمتغيرات الاجتماعية والاقتصادية على خطر الملاريا، وقد أجريت هذه الدراسة لتقييم تأثير العوامل الديموجرافية والوضع الاقتصادي الاجتماعي، والمعرفة والوعي والتثقيف بشأن الإصابة الملاريا.

الأساليب: أجريت دراسة مقطعية في أربعة مراكز صحية تم اختيارها عشوائيا من مركز الرعاية الصحية الأولية في حي أوران أودالجوري، ولاية اسام. تم جمع التركيبة السكانية، والمعرفة الملاريا والمتغيرات الاجتماعية والاقتصادية باستخدام استبيان منظم. وقد تم تحليل ارتباط حدوث الملاريا مع المتغيرات المختلفة باستخدام اختبار مربع كاي. تم تحديد اتجاه الإصابة الملاريا وفقا لفئات الدخل المختلفة، والقرب من المراكز الصحية وعدد من لدغات البعوض في اليوم الواحد أيضا باستخدام اختبار مربع كاي. تم تحديد الخطر النسبي (RR) للمساواة بين الجنسين، ونوع المنزل، ومعرفة واستخدام الناموسيات باستخدام كاتز التقريب.

النتائج: من 71 من أرباب الأسر الذين أجريت مقابلات معهم، كان 70.4 ٪ (71/50) ذكور. وكان متوسط العمر 35 سنة (تراوح بين 20-65 سنة). كان متوسط عدد الأشخاص لكل أسرة خمسة (تراوح بين سنتين إلى 12 سنة). وكان ما يقرب من النصف (54.9 ٪، 71/39) من المشاركين تاريخ من الملاريا في العامين الماضيين، والتي كانت 64.1 ٪ (39/25) ذكور، في حين أن 35.9 ٪ (14/ 39) إناث ($\chi 2 = 5.13$؛ ع = 0.02؛ RR = 1.79، 95٪ CI = 1،2-10،89). من إجمالي السكان الذين شملهم المسح، 46.5 ٪ كانوا فقراء دخلهم الشهري < 2000 روبية هندية (32.14 دولار)، يعيش 49.3٪ في بيوت من الخيزران، ويعيش 35.2 ٪ على مسافة > 3 كم عن أقرب مركز صحي. انخفض عدد المشاركين الذين لديهم تاريخ من الإصابة بالملاريا مع زيادة الدخل الشهري (ع < 0.0001). كانت نسبة الإصابة بالملاريا أعلى بين الأسر التي تعيش في بيوت الخيزران (69.2 ٪)، بالمقارنة مع (20.5 ٪) للمنازل مبنية من الطين و (10.3 ٪) للمنازل المبنية من الخرسانة. وقد لوحظ عدم وجود علاقة ذات دلالة إحصائية بين مستوى التعليم والإصابة بالملاريا (ع = 0.93). وارتبط عدد لدغات البعوض يوميا واستخدام الناموسيات بشكل كبير مع الإصابة بالملاريا بين المشاركين. تفيد تقارير منتظمة حدوث نسبة عالية للإصابة بالملاريا بين المشاركين الذين لم يستخدموا الناموسيات بالمقارنة مع أولئك الذين استخدموا الناموسيات بصفة اليومية (ع < 0.0001).

الاستنتاج: الدخل المنخفض ، نوع المنزل، والمسافة إلى المركز الصحي والمعرفة والوعي حول الملاريا، وعدد لدغات البعوض يوميا واستخدام الناموسيات عوامل ترتبط بشكل إيجابي مع حدوث الملاريا. وزيادة عدد المراكز الصحية القريبة من المناطق الريفية، وتحسين الوضع الاقتصادي وزيادة الوعي حول تدابير الوقاية من الملاريا يساعد على الحد من الإصابة بالملاريا.

Translated from English version into Arabic by Mahmoud Sami, through

TRANSLATORS WITHOUT BORDERS

印度阿萨姆邦基层卫生中心影响疟疾传播的社会经济学因素分析

Kavita Yadav, Sunil Dhiman, Bipul Rabha, PK Saikia, Vijay Veer

摘要

引言：在印度东北部，疟疾的发病率和死亡率较高。由于目前社会和经济学因素对疟疾传播风险的影响分析较少，本研究围绕人口学因素、社会经济学因素、对疟疾了解情况，疟疾防治意识和健康教育等方面进行分析，评估其对疟疾发病的影响。

方法：从阿萨姆邦乌达古里区渔郎郡卫生中心随机选择 4 个二级中心作为研究点，开展横断面调查。通过调查问卷收集人口学数据，疟疾知识知晓情况以及社会经济学数据。采用卡方检验分析疟疾发病与影响因素的关系，不同经济状况、与卫生中心的距离以及每日蚊虫叮咬次数对疟疾发病的影响。通过 Katz 近似法计算不同性别、房子类型、疟防知识知晓情况以及蚊帐使用情况分组的相对风险度（RR）。

结果：调查的 71 位户主中有 70.4%（50/71）是男性。年龄中位数为 35 岁（20～65 岁）。每户人口数的中位数为 5（2～12 个）。一半的调查对象(54.9%，39/71)曾罹患疟疾，64.1% (25/39)为男性，35.9% (14/39)是女性(χ^2=5.13; p=0.02; RR=1.79; 95% CI=1.10–2.89)。调查对象中 46.5%的人月收入低于 2 千卢比（32.14 美元），49.3%居住在竹屋中，35.2%居住地距离最近的卫生保健中心超过 3 千米。月收入和有疟史的人数成反比，月收入增高，有疟史的人数减少（P<0.0001）。居住在竹屋中的调查对象(69.2%)的疟疾发病率高于居住在泥土构造的 Kucha 房子(20.5%)和居住在混凝土建造的 Pucca 房子(10.3%)的人们。受教育水平和疟疾发病无显著相关性（P=0.93）。蚊虫日叮咬次数和蚊帐使用情况与疟疾发病显著相关。未使用蚊帐组的疟疾发病率显著高于使用蚊帐组（P<0.0001）

结论：个人收入、房屋状况、与卫生中心的距离、疟疾防治知晓情况、蚊虫日叮咬次数以及蚊帐使用情况与疟疾发病显著相关。在欠发达地区修建更多的卫生中心，提升当地居民收入以及疟疾防治知晓水平有助于减少疟疾发病。

Translated from English version into Chinese by Zhang Shao-sen, through

CHINESE CENTER FOR DISEASE CONTROL AND PREVENTION
NATIONAL INSTITUTE FOR PARASITIC DISEASES

Facteurs socio-économiques déterminants dans le risque de transmission du paludisme dans un centre de santé endémique principal à Assam, Inde

Kavita Yadav, Sunil Dhiman, Bipul Rabha, PK Saikia, Vijay Veer

Résumé

Contexte : Le paludisme constitue une cause majeure de morbidité et de mortalité dans le nord-est de l'Inde. Étant donné le peu d'informations disponibles sur l'influence possible des facteurs socio-économiques sur le risque de transmission paludique, la présente étude a été réalisée pour évaluer l'influence des facteurs démographiques, du statut socio-économique, des connaissances, de la sensibilisation et de l'éducation sur l'apparition du paludisme.

Méthodes : Une étude transversale a été menée dans quatre centres de santé secondaires, sélectionnés au hasard, dépendants du centre de santé principal d'Orang dans le district d'Udalguri, à Assam. Un questionnaire structuré a été utilisé pour recueillir les données sur la démographie, la connaissance du paludisme et les variables socio-économiques. L'association de l'apparition du paludisme et des différentes variables a été analysée à l'aide d'un test du chi carré. Les tendances d'apparition du paludisme selon différents groupes de revenus, la proximité des centres de santé et le nombre de piqures de moustiques par jour ont également été déterminées à l'aide du test du chi carré. Le risque relatif (RR) selon le sexe, le type d'hébergement, la connaissance et l'utilisation de moustiquaires a été déterminé à l'aide de l'approximation de Katz.

Résultats : Sur les 71 chefs de ménage interrogés, 70,4 % (50/71) étaient des hommes. L'âge moyen était de 35 ans (plage allant de 20 à 65 ans). Le nombre moyen de personnes par ménage était de cinq (plage allant de deux à 12). Environ la moitié (54,9 %, 39/71) des participants avait déjà contracté le paludisme au cours des deux dernières années. Parmi ceux-ci 64,1 % (25/39) étaient des hommes et 35,9 % (14/39) étaient des femmes (χ^2=5,13 ; p=0,02 ; RR=1,79 ; 95 % CI=1,10–2,89). Sur l'ensemble de la population interrogée, 46,5 % étaient pauvres avec un revenu mensuel de <2 000 INR (32,14 $), 49,3 % vivaient dans des maisons en bambou et 35,2 % vivaient à une distance de >3 km du centre de santé le plus proche. Le nombre de participants ayant déjà contracté le paludisme diminuait selon l'augmentation du revenu mensuel (p<0,0001). L'apparition du paludisme était plus élevée parmi les ménages vivant dans des maisons en bambou (69,2 %), par rapport aux maisons

Kucha (20,5 %) faites en boue et aux maisons Pucca (10,3 %) faites en béton. Aucune association significative n'a été établie entre le niveau d'éducation et l'apparition du paludisme (p=0,93). Un lien a été établi entre le nombre de piqures de moustiques par jour et l'utilisation de moustiquaires, et l'apparition du paludisme chez les participants. L'infection paludique était plus élevée chez les participants n'utilisant pas de moustiquaires par rapport à ceux utilisant des moustiquaires chaque jour (p<0,0001).
Conclusion : Un revenu plus faible, le type d'hébergement, la proximité d'un centre de santé secondaire, les connaissances et la sensibilisation au paludisme, le nombre de piqures de moustiques par jour et l'utilisation de moustiquaires ont été associés de façon positive à l'apparition du paludisme. L'augmentation du nombre de centres de santé secondaires proches des zones rurales, l'amélioration du statut économique et de la sensibilisation aux mesures de prévention contre le paludisme aideront donc à réduire la morbidité associée au paludisme.

Translated from English version into French by Corinne Vasseur, through

TRANSLATORS
WITHOUT BORDERS

Социально-экономические детерминанты для риска передачи малярии в эндемичном Центре медико-санитарной помощи в штате Ассам в Индии.

Кавита Ядав, Сунил Дхиман, Бипул Рабха, ПК Сайкия, Виджей Виир

Краткое изложение

История вопроса: Малярия является главной причиной заболеваемости и смертности в Северо-восточной Индии. Так как количество информации о потенциальном воздействии социально-экономических переменных на риск малярии ограничено, данное исследование проводилось с целью оценки влияния демографических факторов, социально-экономического статуса, а также знаний, осведомленности и образования на заболеваемость малярией.

Методы: Было проведено одномоментное межгрупповое исследование в четырех произвольно выбранных субцентрах Орангского центра медико-санитарной помощи в округе Удалгури, штате Ассам. Демографические данные, знания о малярии и социально-экономические переменные собирались с помощью специально разработанного вопросника. Связь между заболеваемостью малярией и различными переменными анализировалась с помощью теста хи-квадрата. Тенденция заболеваемости малярией для различных групп по уровню доходов, близости к медицинским центрам и количеству москитных укусов в день также определялась с помощью теста хи-квадрата. Относительный риск (ОР) по полу, виду дома, знаниям и использованию надкроватных москитных сеток определялся с помощью приближенной формулы Каца.

Результаты: Из 71 опрошенных глав семей 70,4% (50/71) были мужчины. Средний возраст – 35 лет (в диапазоне от 20 до 65 лет). Среднее количество членов семьи составило 5 человек (от 2 до 12). Около половины (54,9%, 39/71) участников болели малярией в последние два года, из которых 64,1% (25/39) были мужчины, а 35,9% (14/39) – женщины (χ^2=5,13; p=0,02; RR=1,79; 95% CI=1,10–2,89). Из всего рассмотренного населения 46,5% были бедными с месячным доходом <2 000 INR (32,14 $), 49,3% жили в бамбуковых хижинах, а 35,2% жили на расстоянии >3 км от ближайшего медицинского центра. Количество участников, болевших в прошлом малярией, уменьшалось с ростом месячного дохода (p<0,0001). Заболеваемость малярией была выше в семьях, проживающих в бамбуковых хижинах (69,2%), по сравнению глиняными

домами Куча (20,5%) и бетонными домами Пукка (10,3%). Между уровнем образования и заболеваемостью малярией не было обнаружено значительной связи (p=0,93). Количество москитных укусов в день и использование надкроватных сеток были значительно связаны с заболеваемостью малярией среди участников. Участники, не пользовавшиеся регулярно надкроватными сетками, сообщали о более частой заболеваемости малярией по сравнению с теми, которые ежедневно пользовались сетками ($p<0.0001$).

Заключение: Низкий доход, виды домов, расстояние до ближайшего медицинского субцентра, знания и осведомленность о малярии, количество москитных укусов в день и использование надкроватных сеток были безусловно связаны с заболеваемостью малярией. Увеличение количества медицинских субцентров в сельской местности, улучшение экономического статуса и повышение осведомленности о мерах предотвращения малярии помогут, таким образом, сократить связанную с малярией смертность.

Translated from English version into Russian by Elena McDonnell, through

TRANSLATORS
WITHOUT BORDERS

Determinantes socio-económicos para el riesgo de transmisión de malaria en un centro primario de salud en Assam, India

Kavita Yadav, Sunil Dhiman, Bipul Rabha, PK Saikia, Vijay Veer

Abstracto

Antecedentes: La malaria es una causa mayor de morbidez y mortalidad en el noreste de India. Como hay información limitada disponible sobre la potencial influencia de variables socio-económicas sobre el riesgo de malaria, el actual estudio fue realizado para evaluar la influencia de factores demográficos, el estatus socio-económico, y el conocimiento, la consciencia y la educación sobre la ocurrencia de malaria.

Métodos: Un estudio inter-seccional fue realizado en cuatro sub-centros de salud seleccionados aleatoriamente del centro primario de salud de Orang en el distrito de Udalguri, Assam. Factores demográficos, conocimiento de malaria y variables socio-económicas fueron recolectados usando un cuestionario estructurado. La asociación de ocurrencia de malaria con diferentes variables fue analizada usando una prueba de ji-cuadrado. La tendencia de la ocurrencia de malaria para los diferentes grupos de ingresos, proximidad a los centros de salud y el número de picadas de mosquito por día también fue determinada usando la prueba ji-cuadrado. El riesgo relativo (RR) según género, tipo de casa, conocimiento y uso de redes para camas fue determinado usando la aproximación Katz.

Resultados: De los 71 jefes de hogar entrevistados, 70,4% (50/71) eran hombres. La edad media era de 35 años (rango de 20 a 65 años). El número medio de personas por hogar era cinco (rango de dos a 12). Aproximadamente la mitad (54,9%, 39/71) de los participantes tenía una historia de malaria en los últimos dos años, de los cuales el 64,1% (25/39) eran hombres, mientras que 35,9% (14/39) eran mujeres (χ^2=5,13; p=0,02; RR=1,79; 95% CI=1,10–2,89). Del total de la población encuestada, 46,5% era pobre con un ingreso mensual menor a 2000 INR (32,14 $), 49,3% vivía en casas de bambú y 35,2% vivía a una distancia mayor a 3 km del centro de salud más cercano. El número de participantes que tenía una historia de malaria disminuyó con un ingreso mensual creciente (p<0,0001). La ocurrencia de malaria fue más alta entre los hogares que vivían en casas de bambú (69,2%), en comparaciones con las casas de Kucha (20,5%) construidas de barro y las casas de Pucca (10,3%) hechas de concreto. No se observó asociación significativa entre el nivel de educación y la ocurrencia de malaria (p=0,93). El número de picadas de mosquito

por día y el uso de redes para camas estuvieron significativamente asociados a la ocurrencia de malaria entre los participantes. Los participantes que no usaban redes para cama regularmente reportaron una mayor ocurrencia de infección de malaria en comparación con aquellos que usaban redes para camas todos los días ($p<0,0001$).

Conclusión: El menor ingreso, el tipo de casa, la distancia a un sub-centro de salud, el conocimiento y la consciencia sobre la malaria, el número de picadas de mosquitos por día y el uso de redes para camas fueron positivamente asociados a la ocurrencia de malaria. Aumentar el número de sub-centros de salud cerca de áreas rurales, mejorar el estatus económico e incrementar la consciencia sobre las medidas de prevención de la malaria, por lo tanto, ayudarán a reducir las morbideces asociadas a la malaria.

Translated from English version into Spanish by Denise Tarud, through

TRANSLATORS WITHOUT BORDERS
